# Cobalt Ion Removal by Activated Carbon and Biochar Derived from *Sargassum* sp.

**DOI:** 10.3390/ijms26167666

**Published:** 2025-08-08

**Authors:** Julie Mallouhi, Emőke Sikora, Kitti Gráczer, Olivér Bánhidi, Sarra Gaspard, Marckens Francoeur, Yeray Alvarez-Galvan, Francesca Goudou, Béla Viskolcz, Emma Szőri-Dorogházi, Béla Fiser

**Affiliations:** 1 Institute of Chemistry, University of Miskolc, 3515 Miskolc-Egyetemváros, Hungary; 2 Higher Education and Industrial Cooperation Centre, University of Miskolc, 3515 Miskolc-Egyetemváros, Hungary; 3 Laboratory COVACHIM-M2E, EA 3592, Université des Antilles, BP 250, 97157 Pointe à Pitre Cedex, France; 4 Department of Biology and Chemistry, Ferenc Rakoczi II Transcarpathian Hungarian College of Higher Education, 90200 Beregszász, Ukraine; 5 Department of Physical Chemistry, Faculty of Chemistry, University of Lodz, 90-236 Lodz, Poland

**Keywords:** activated carbon, biochar, *Sargassum* sp., adsorption of Co ions, adsorption isotherm

## Abstract

Activated carbon (AC) and biochar (BC) are porous substances derived from any carbonous material known to be highly effective adsorbents, making them valuable for removing pollutants like heavy metals. This study evaluated and compared the potential of AC and BC produced from *Sargassum* sp. by chemical activation and pyrolysis process for heavy metal removal, specifically Co^2+^ ions, to commercial AC (COMAC). Various techniques were employed to characterize these samples including FTIR, zeta potential, and surface area. Additionally, considering parameters such as pH, initial solution concentration, and the effect of AC/BC dose were investigated. The adsorption isotherm was also assessed. The results showed that a strong dependence of the adsorption capacity on pH was observed with optimal performance at ~6.8. Additionally, the optimal initial solution concentration was determined to be ~2 mmol/L. According to the Langmuir isotherm model, AC derived-*Sargassum* sp. exhibited maximum uptakes of 468.97 mg/g, higher than COMAC and BC. The experiment at different adsorbent dosages revealed that AC from *Sargassum* sp. outperformed other samples, with adsorption capacity observed at 94.94% as the dosage increased.

## 1. Introduction

Water is one of the most crucial resources on Earth, responsible for the survival of all living organisms [1]. The accelerated rate of industrialization has significantly exacerbated the issue of water pollution [2,3]. Toxic metals are toxic pollutants that enter surface and groundwater through various activities, including industrial processes, mining, and agriculture [4,5]. Toxic metals provide a significant environmental risk due to their toxicity and availability. Although certain metals are essential in trace amounts, exceeding their tolerance levels can lead to various diseases, posing significant risks to both human and environmental health [1,2,6]. The removal of hazardous heavy metal pollutants from water and wastewater is one of the most critical environmental challenges currently under investigation [7]. Various techniques, such as adsorption, chemical precipitation, electrochemical precipitation, chemical oxidation, ion exchange, and membrane filtration, are employed for this purpose, which vary in their effectiveness and cost [7]. Among these, adsorption is considered one of the most effective methods due to its high removal efficiency, low cost, ease of operation, and the ability to regenerate adsorbents through an appropriate desorption process [8].

**Figure 1:**
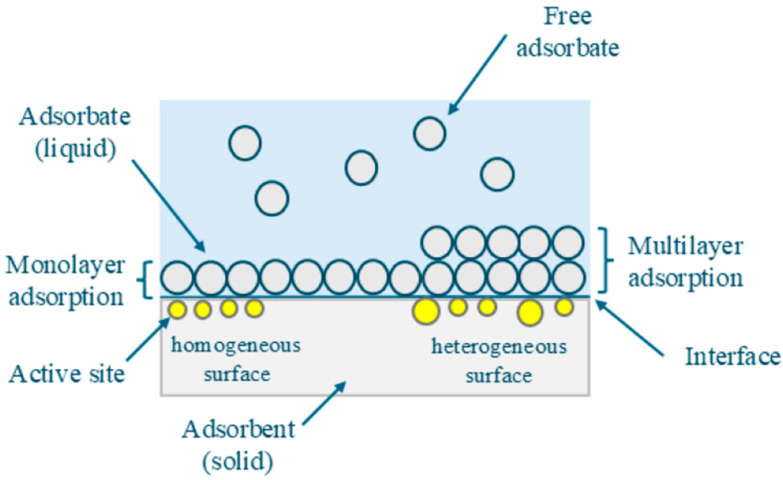
Schematic representation of the adsorption process.

The adsorption process refers to the accumulation of substances or particles at the interface between a solid surface and a surrounding medium, such as a liquid or gas, where the material to be removed is called ‘adsorbate’, while the material utilized for adsorption is known as ‘adsorbent’. In other words, the adsorption process involves the formation of a layer of adsorbate, such as metal ions, on the surface of the adsorbent (Figure 1) [9,10].

The adsorption process can be classified as either physical adsorption (physisorption) or chemical adsorption (chemisorption), based on the nature of the interaction [6]. Physisorption occurs through van der Waals forces, forming a multilayer of adsorbate, while chemisorption involves strong chemical bonds, such as ionic or covalent, resulting in a monolayer of adsorbate [6,10] and leads to the formation of a monolayer of adsorbate on the adsorbent [11].

To understand and employ the adsorption processes, particularly in environmental remediation and wastewater treatment facilities, adsorption isotherms are crucial and can provide insights into the maximum adsorption capacity, which is a key factor in assessing the performance and effectiveness of adsorbents. Isotherm refers to the relationship between the equilibrium adsorbate concentrations in the liquid phase and the equilibrium adsorption amount in the solid phase at a certain temperature [10]. Several isotherm models exist for analyzing experimental adsorption equilibrium parameters, such as Langmuir, Volmer, Brunauer–Emmett–Teller, Aranovich model, Freundlich, Redlich-Peterson, Temkin, Toth, and Sips.

Although several models also exist, the Langmuir and Freundlich isotherms are the most commonly used ones to describe the adsorption of metal ions, dyes, as well as other types of organic pollutants onto adsorbents [12,13]. These two models are commonly employed in adsorption studies due to their applicability. Both models effectively describe the relationship between q_e_ (the quantity adsorbed at equilibrium, mg/g) and C_e_ (the concentration of adsorbate ions remaining in the bulk solution at equilibrium, mg/L) [14]. In general, experimental data are fitted to different adsorption isotherm models, and the best-fitting model is used to define equilibrium adsorption. After selecting an appropriate adsorption model, the model’s adsorption parameters were obtained and utilized to characterize the properties of the process [15].

Key adsorption parameters such as pH, initial metal concentration, and adsorbent dose should also be considered, as these are the most important parameters influencing the efficiency and performance of the adsorption process [6]. Furthermore, electrostatic interactions between positively charged metal ions and negatively charged functional groups on the surface of adsorbents can play a significant role in enhancing the adsorption capacity [10].

The key criteria for an adsorption process are the adsorbent’s selectivity, high adsorption capacity, and low cost [16]. Activated carbon (AC) and biochar (BC) have been shown to be effective adsorbents for the removal of a wide range of contaminants that exist in the aquatic environment, due to their high surface area and porosity, in addition to a variety of surface functional groups like carboxyl (-COOH), and hydroxyl (-OH), which play a role in adsorbing contaminants [7,17,18]. These functional groups attract heavy metal ions either through electrostatic attraction or surface metal complexation [1].

In Guadeloupe, there is an issue of invasive *Sargassum* sp. in huge quantities on the shores, which poses a significant environmental challenge. However, this issue can be turned into an opportunity by using *Sargassum* sp. as a raw material for activated carbon (AC) production [19,20,21,22].

Activated carbon derived from the macroalga *Sargassum* sp. has shown significant potential as a biosorbent material. Studies have demonstrated its effectiveness in water treatment applications, particularly for removing metals from water [2]. When tested in a synthetic multi-solute system, the activated carbon from *Sargassum* sp. was evaluated for its adsorption capacity for four metal ions lead, copper, zinc, and manganese, and the optimal adsorption conditions were found to include a pH of 6, a biosorbent dosage of 3 g/L, and an equilibrium time of 50 min. Under these conditions, *Sargassum* sp. achieved maximum uptake values of 44.2 mg/g for lead, 17.7 mg/g for copper, 7.3 mg/g for zinc, and 5.7 mg/g for manganese [23]. In some studies, the adsorption capacity of AC materials for potentially toxic metals has been investigated, and it was shown that the maximum amount of Cu (II) that could be removed using phosphoric acid-activated rice husk at pH 4 was 17.0358 mg/g at an adsorbent dosage of 2 g/L [24]. The activated carbon from apricot stone with H_3_PO_4_ and its ability to remove Co^2+^ was studied as well, and it was observed that the maximum adsorption capacity from the Langmuir model is found to be 111.11 mg/g at pH 9 [25].

Also, several biochars derived from agricultural wastes were tested in adsorption tests [26] and it was found in a comparative study that biochar employed for heavy metal adsorption was more efficient than commercial activated carbon for Cu^2+^ ion removal, where BC was made from peanut, canola, and soybean straw processed at 400 °C [27].

In other studies, the application of coconut fiber biochar for effective removal of Co (II) ions from synthetic wastewater was evaluated. The maximum monolayer adsorption capacity was determined to be 106.80 mg/g [28]. However, in another study, the removal of cobalt ions from aqueous solutions by biochar derived from Greenhouse Crop Residue was lower, with an adsorption capacity of 28.8 mg/g [29]. Thus, biochar is applicable to remove potentially toxic metals from contaminated water bodies.

Cobalt is increasingly present in wastewater due to industrial activities like metallurgical, mining, and paint. Its potential toxicity to aquatic life and human health makes its removal a critical environmental concern [25].

While many studies have investigated metal adsorption using biochar and activated carbon from various sources, few have explored the use of *Sargassum* sp. for cobalt ions removal.

The main objective of this study is to test the applicability of carbonaceous materials like activated carbon, biochar derived from *Sargassum* sp., samples as water treatment agents for the removal of cobalt ions from aqueous solutions.

## 2. Results and Discussion

### 2.1. Characterization of the AC/BC Samples

FTIR analysis showed that all samples contained identical functional groups, specifically carbonyl and hydroxyl groups, on the surface of carbonaceous material (AC/BC) (Figure S1). In the case of the surface area measurement, the results showed that activated carbon derived from *Sargassum* sp. (AC) exhibited the highest surface area, measuring 1695 ± 7 m^2^/g (a higher surface area suggests a more porous structure, which improves the efficacy of materials for adsorbing potentially toxic metals from water). While the zeta potential measurements revealed that the biochar had the most negative values, −22.80 mV ± 0.28, indicating superior dispersion stability compared to COMAC and AC, which displayed zeta potentials of −13.21 ± 0.43 mV and −20.07 ± 1.71 mV, respectively (Table 1) [30].

### 2.2. Adsorption Test

#### 2.2.1. Influence of pH

The pH_pzc_ was determined for the COMAC, AC, and BC samples. Estimating the pH_pzc_ is crucial, as it influences the adsorption behavior of the materials. When the pH of the solution is lower than the pH_pzc_, the surface of the porous materials becomes a bit more positively charged, and thus, the anion adsorption is favored. Conversely, if the solution pH is higher than the pH_pzc_, the surface of the samples acquires a bit more negative charge, enhancing the attraction of cation adsorption. The pH_pzc_ of the COMAC, AC, and BC were 6.98 ± 0.01, 3.40 ± 0.01, and 6.50 ± 0.01, respectively.

**Figure 2:**
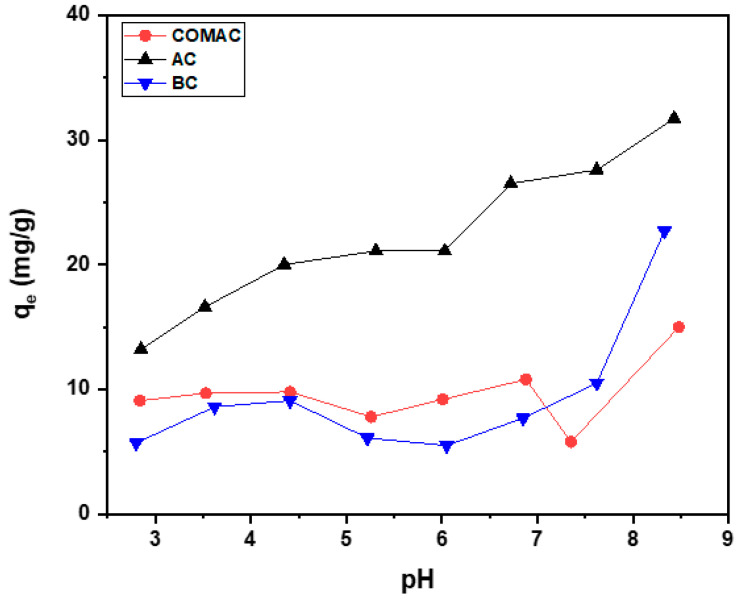
Effect of the pH of the solution on the adsorption of Co^2+^ by 0.05 g of the three samples studied, COMAC, AC, and BC, at 2 mmol/L initial concentration.

Given the influence of pH on adsorption mechanisms, it is essential to assess how surface charge variations affect metal ion removal. At low pH, certain functional groups may become protonated, which reduces their ability to provide negatively charged active sites, thereby preventing the formation of complexes between Co(II) ions and the other functional groups present on activated carbon or biochar surfaces [31]. The Co(II) removal at strong acidic pH values was found to be small in a range of 5.7–13.2 mg/g (5.48–12.62%) (Figure 2, Table 2). However, the adsorbed amount of cobalt ions increases consistently with increasing pH values, and this is noticed clearly in the case of a sample of activated carbon derived from *Sargassum* sp., which has a better uptake capacity compared to the biochar sample at different pH. It was found that pH = 6.8 is the optimum value, in which the Co(II) removal was the highest for sample AC with an adsorbed amount of 26.5 mg/g and removal efficiency of 25.25% which is higher than the adsorbed amount by COMAC and biochar BC with removal efficiency 10.37% and 7.41%, respectively, at the same pH.

The observed results align with the pH_pzc_ values of the materials. Since AC has a low pH_pzc_ of 3.40, its surface remains negatively charged at pH 6.8, favoring Co(II) cation adsorption, leading to higher adsorption. On the other hand, COMAC and BC, with higher pH_pzc_ values of 6.98 and 6.50, respectively, exhibit less favorable surface charges for Co(II) removal for cation attraction, resulting in lower adsorption capacities.

The adsorption of this metal at pH > 6.8 is related to the formation of metal hydroxide species, such as soluble Co(OH)^+^ and/or the formation of insoluble precipitate of Co(OH)_2_ [31]. In this case, the metal ions are removed from the solution due to the formation of Co(OH)_2_ and not due to the adsorption of free Co(II) ions. Thus, the pH has to be kept at pH = 6.8 to avoid precipitation.

It can be concluded that activated carbon and biochar samples display varying capacities for adsorbing metal ions. Previous measurements indicate that the surface zeta potential of all samples ranges between −22.80 mV and −13.21 mV, indicating a negative charge on the surface of the COMAC, AC, and BC particles. Consequently, these materials probably adsorbed metal ions by electrostatic interaction between positively charged metal ions and the negatively charged adsorbents. This electrostatic interaction likely contributes to the enhanced adsorption of metal ions as pH increases. Additionally, all samples contain surface functional groups such as -COOH and -OH, which can interact with metal ions to form surface complexes on both activated carbon and biochar. By increasing pH, more of these functional groups will be deprotonated, thereby these negatively charged groups will further enhance the possibility of complex formation with metal ions [10,25,31,32].

Similar results were presented previously in some experiments, where activated carbon derived from pyrolyzed potato peels was utilized to remove cobalt ions [31]. Chemical activation was achieved by treating the material with phosphoric acid at temperatures of 400 °C, 600 °C, and 800 °C for 2 h. The study revealed that as the pH increased from 2 to 5, the percentage of removal also increased across all temperatures. At pH = 6, the highest cobalt uptake was recorded, reaching 85.7% by employing the AC prepared at 400 °C and 92% by using samples prepared at 600 °C, while the adsorption capacities were 373 mg/g and 405 mg/g, respectively [31].

#### 2.2.2. Effect of Initial Cobalt Concentration and Adsorption Isotherm Investigation

The removal efficiency percentage of metal ions is directly affected by their initial concentration in the solution [11]. At low concentrations, metals are adsorbed onto specific sites. As the concentration increases, all adsorption sites become occupied, and the adsorbent surface may become covered by the initially adsorbed metal ions [33]. Higher initial metal ion concentrations provide a strong driving force between the liquid and solid phases [34]. The results showed that the adsorption capacity increased with increasing initial concentration for all the adsorbents. This is attributed to the high initial concentration increases the driving force for the mass transfer of metal ions onto the adsorbent’s surfaces and more efficient utilization of the active sites on the adsorbent, thereby enhancing the efficiency of the adsorption process [11,35]. Based on the results, the optimal metal concentration was ~2 mmol/L.

**Figure 3:**
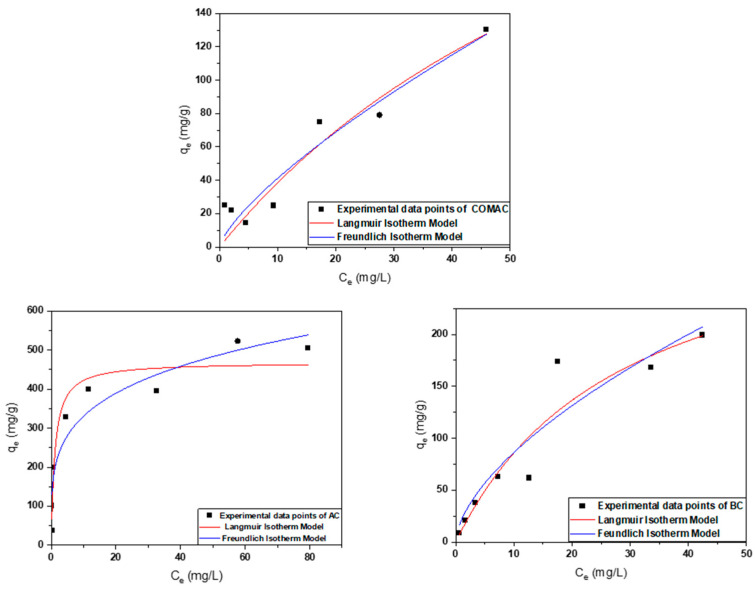
Adsorption isotherms fitted to the Co^2+^ adsorption data measured by using different adsorbents.

The study of concentration effects plays a crucial role in improving the accuracy of isotherm results. The adsorption isotherms provide essential data for understanding the adsorption behavior [36]. The two models of Langmuir and Freundlich were fitted to the experimental adsorption isotherms data of Co^2+^ onto the adsorbent surface at pH 6.8 and different initial concentrations, and the obtained results are shown in (Figure 3 and Table 3).

The Langmuir isotherm, which assumes monolayer adsorption on a surface, provided insights into the maximum adsorption capacities of the tested adsorbents. The maximum monolayer adsorption capacities estimated by the Langmuir isotherm were 468.97, 361.23, and 334.36 mg/g for AC, COMAC, and BC, respectively. Among these, the sample AC derived from *Sargassum* sp. exhibited better capacity and greater potential to adsorb cobalt ions at saturation than biochar and commercial AC, where high q_m_ and a steep initial isotherm slope indicate strong favorability (as the initial concentration increases, the available sites may become saturated, and adsorption reaching a plateau) as shown in (Figure 3). Additionally, the Langmuir constant K_L,_ which reflects the affinity between the adsorbent and the cobalt ions, was also higher for AC with values of 0.9 (L/mg). These higher values suggest a stronger interaction between cobalt ions and the adsorbent surfaces, indicating that these adsorbents may be more efficient in removing cobalt ions from the solution. The Langmuir isotherm applied to the data with a correlation coefficient (R^2^) of ~0.9 for all the samples, indicating that Co^2+^ adsorption likely occurred on a homogeneous surface through monolayer adsorption, with no interaction between the adsorbed molecules [37,38].

The Freundlich isotherm was also applied, which describes the multilayer adsorption on a heterogeneous surface primarily driven by physisorption. The R^2^ values for BC are close to 1 (Table 3), indicating favorable adsorption and strong interaction between Co^2+^ ions and the functional groups on the adsorbent surface. The Freundlich model shows a better fit compared to the Langmuir model, though no plateau is observed, indicating incomplete surface coverage due to insufficient metal ion concentration. All in all, it can be concluded that although the BC adsorption data fits well to the Freundlich model, the lack of a clear saturation point and lower overall uptake may suggest partial surface coverage, possibly influenced by particle aggregation, which could reduce the effective surface area available for multilayer adsorption. However, high K_F_ and n values were observed for AC, indicating a high binding capacity and strong affinity between these adsorbents and Co^2+^ ions [23].

All in all, the significantly high surface areas of AC (1695 m^2^/g) contribute directly to their high adsorption capacities, as higher surface areas provide more active sites for adsorbate interactions. This increased availability of adsorption sites allows these materials to capture and retain more cobalt ions than samples with lower surface areas, COMAC (1120 m^2^/g) and BC (854 m^2^/g). In addition to that, the actual uptake is also influenced by other key factors such as the point of zero charge (pH_pzc_), which plays a critical role in determining surface charge under experimental pH conditions. As previously mentioned, AC with a low pH_pzc_ of 3.4, which results in a strongly negatively charged surface at pH 6.8, enhances electrostatic interactions with Co^2+^ ions.

#### 2.2.3. Effect of Adsorbent Dosage

**Figure 4:**
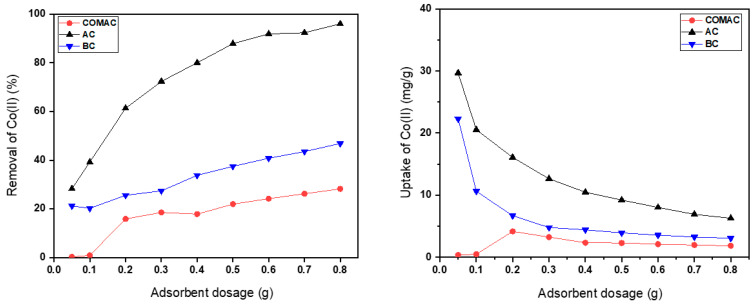
Effect of adsorbent dosage on adsorption of Co^2+^ ions (pH = 6.8, initial concentration = 2 mmol/L, agitation rate = 210 rpm, contact time = 1 h).

The influence of adsorbent dosage on the adsorption of metal ions was examined by varying dosages from 0.05 to 0.8 g while the initial cobalt concentration was held constant at 2 mmol/L at room temperature, and pH was kept at ~6.8 (Figure 4).

The results showed that the metal uptake (the amount of cobalt ions adsorbed per unit mass of the adsorbent) decreases by increasing the adsorbent dosage. At lower adsorbent doses, the concentration of cobalt ions to the available adsorption sites is higher, enabling the most active sites on the adsorbent to be occupied. However, at higher adsorbent dosage, the available cobalt ions become insufficient to occupy all exchangeable adsorption sites, leading to a reduction in metal uptake per unit mass of the adsorbent. Additionally, increasing the adsorbent dosage can also cause adsorbent particle overlap, potentially interfering with binding sites. This interference may further contribute to the observed decrease in specific metal uptake [23].

On the contrary, it was observed that the removal efficiency (percentage of metal ions removed from the solution) increased with an increase in adsorbent dosage. This is attributed to the greater overall surface area provided by the higher amount of adsorbent, which increases the number of available binding sites for adsorption [9,23,39].

The results showed that the AC sample was the most effective adsorbent for removing Co^2+^ ions compared to COMAC and BC at all dosages. This superior performance can be attributed to their higher surface area, which provides more active sites for adsorption. The removal efficiency of AC exhibited an increase in removal efficiency, from 28% to 94.94% as the dosage increased.

Several studies observed this trend previously, where the adsorbent from *Sargassum* sp. was used to adsorb four metal ions, Pb^2+^, Cu^2+^, Zn^2+^, and Mg^2+^ from a synthetic multi-solute system and real stormwater runoff and it was observed that the metals removal efficiency increased with increasing adsorbent amount whereas metals uptake capacity decreased at higher adsorbent dosages [23].

In contrast, biochar showed significantly lower efficiency for Co^2+^ ion removal than AC (Figure 4). For COMAC, even at higher dosages, the removal efficiency remained limited, reaching only 28%.

The optimal dosage for COMAC would be approximately 0.2 g, as increasing the dosage beyond this point does not yield a significant increase in removal efficiency. For AC and BC samples, the optimal adsorbent dose would be chosen based on maximum removal efficiency. In such cases, adding more adsorbent can further enhance the efficiency of the removal of metal ions, as can be seen in the case of AC and BC samples, where the removal efficiency increases steadily with dosage. The differences in adsorption capacities among the various adsorbent dosages could be attributed to the type of surface functional groups involved in the adsorption of metal ions from the solution [36].

Overall, AC demonstrated its suitability for high-efficiency cobalt removal, whereas BC, despite its lower performance, could still be considered for applications.

These findings highlight the adsorption capacity of AC/BC derived from *Sargassum* sp. for metal ion removal, particularly for cobalt. Several studies have explored the effectiveness of activated carbon and biochar from various natural sources in removing potentially toxic metals from water (Table 4). When compared to AC/BC from other sources, the AC/BC derived from *Sargassum* sp. demonstrated significantly higher or similar cobalt adsorption capacity, highlighting its potential as an efficient adsorbent for heavy metal removal (Table 4).

## 3. Materials and Methods

### 3.1. Materials

Three different samples were tested and compared: commercial activated carbon (COMAC), Sigma-Aldrich (France) prepared from coconut by physical activation with CO_2_ as the activating gas, activated carbon (AC) prepared from *Sargassum* sp. by chemical activation with phosphoric acid, and biochar prepared from *Sargassum* sp. (elaborated using microwaves). The samples were identified as COMAC, AC, and BC, respectively.

The *Sargassum* sp. (*Sargassum natans* and *Sargassum fluitans*) was collected from La Datcha beach in Guadeloupe and was treated before being used as a base material for producing activated carbon and biochar [20].

From this material, the activated carbon sample was produced by chemical activation where the *Sargassum* sp. was soaked with 85% phosphoric acid (H_3_PO_4_) (3:1 mass ratio) for 15 h (as the concentration of phosphoric acid increases, the porosity and surface area of the material significantly improved) [18], then heated to 600 °C at 5 °C/min, held for two hours, and cooled naturally. Nitrogen gas was supplied to the furnace at a rate of 80 mL/min throughout the entire process. At the end of the process of the AC, it was washed with deionized water in a stirred baker at a temperature of 80 °C. This washing was repeated twice to ensure that the pores of the AC were completely free of phosphoric acid and other potential contaminants.

*Sargassum* sp. are regularly characterized before AC preparation, and they are shown to contain heavy metals such as Arsenic, and some of the work has shown that pre-treatments can be applied to reduce their concentration. Before adsorbent preparation, the arsenic content in *Sargassum* sp. was about 118 µg/kg, and this amount was reduced to 66 µg/kg after biosorbent preparation.

On the other hand, biochar was produced from *Sargassum* sp. as well by the pyrolysis process by using a microwave oven at (~650 °C) for 15 min under an inert nitrogen atmosphere. It was then washed with 5 M HCl and deionized water to pH 7, followed by drying [30,49].

### 3.2. Characterization of Carbonaceous Material

All samples were analyzed to assess their structure and functionalities. Zeta potential measurements evaluated the surface charge of the activated carbon and biochar, which was determined by using a Litesizer DLS 500 particle analyzer (Graz, Austria), while a Fourier-transform infrared spectroscopy (FTIR) spectrometer identified surface functional groups, which were determined by using a Bruker Vertex 70 FTIR spectrometer (Ettlingen, Germany). The specific surface area was also measured using nitrogen (N_2_) adsorption studies at 77 K by using a Micromeritics ASAP 2020 apparatus (Mérignac, France). The specific surface area was determined using a linear fit of the Brunauer–Emmett–Teller (BET) equation within the relative pressure range (P/P_0_) of 0.103 to 0.225 [30].

### 3.3. Adsorption Studies

Various parameters were studied to determine the optimal conditions in the adsorption test, such as pH, initial concentration of the solution, and the amount of the dosage. The adsorption isotherm was evaluated as well.

#### 3.3.1. Effect of pH Solution

The pH of the aqueous solution is an important parameter in adsorption [50]. During this process, the binding of metal ions to the adsorbent is often influenced by factors such as the functional groups, surface charges, degree of ionization, and solubility of the adsorbent [34,51], and electrostatic interactions between metal ions and adsorbent surface groups, which are affected by pH. To enhance adsorption, the pH of the solution must be adjusted so that the metal ions and adsorbent surface groups are of opposite charge. This will increase adsorption by promoting strong electrostatic interactions [52].

Given the significant role of pH in adsorption, it is essential to determine the point of zero charge (pH_pzc_) before conducting adsorption experiments, as pH_pzc_ defines the linear range of pH sensitivity. This property provides insights into the surface polarity, which gives information on its adsorption capacity for ionic species. To determine the pH_pzc_ of all carbonaceous samples, 0.1 g of carbonaceous material was added to 20 mL of a 0.1 M NaCl solution with initial pH values ranging from 2 to 13, adjusted using 0.5 M NaOH or HCl. Then, the samples were agitated at 150 rpm for 24 h at 25 °C. The initial and final pH values were then determined to calculate the pH_pzc_ [33].

**Figure 5:**
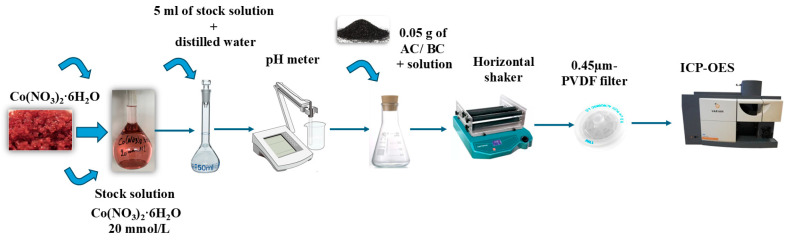
The workflow of the adsorption tests employed during the study.

For the adsorption test, the stock solution was prepared by dissolving the reagent, 1.455 g of cobalt (II) nitrate hexahydrate (Co(NO_3_)_2_·6H_2_O), in volumetric flasks and diluted to 250 mL with distilled water (was prepared with reverse osmosis (RO) with an initial concentration of 20 mmol/L. Then, 5 mL of stock solution was pipetted and diluted in a 50 mL flask with distilled water, where the pH values were adjusted to the range ~2.0–8.0 by 1 M HCl and 1 M NaOH and measured using a calibrated digital pH meter (VWR pHenomenal^®^ pH meter), and the initial concentration of the solution was 2 mmol/L in a 50 mL flask. The experiments were carried out using 9 Erlenmeyer flasks, each containing 0.05 g of carbonaceous material in 50 mL of diluted cobalt solution. After that, these flasks were shaken in a horizontal shaker for 1 h at 210 rpm at room temperature. After shaking, the solutions were filtered by using a syringe filter (0.45 µm-PVDF filter). The final concentration of metal ions was measured by using an inductively coupled optical emission spectrometer (ICP-OES, Varian 720 ES, Lab-EX, (Budapest, Hungary)) (Figure 5).

The amount of metal ions adsorbed onto the carbonaceous materials (AC or BC) was calculated using the following equation:

(1) $$q_{e}=\frac{\left(C_{i}-C_{e}\right)\times V}{m}$$

where *q_e_* is the amount of metal adsorbed by the adsorbent (mg/g), *C_i_* is the initial metal concentration (mg/L), *C_e_* is the equilibrium metal concentration after filtration (mg/L), *V* is the volume of the initial solution (L), and *m* is the weight of carbonaceous material (g).

The percentage removal of metal ions was calculated using the following formula:

(2) $$Removal\;\%=\frac{C_{i}-C_{e}}{C_{i}}\;\times 100$$

#### 3.3.2. Effect of the Initial Metal Concentration

The adsorption capacity of the adsorbents is affected by the metal concentration. The initial concentration of metal ions can impact the efficiency of metal removal by affecting the availability of specific surface functional groups and their ability to bind metal ions effectively [51].

To assess the effect of initial ion concentration on equilibrium, various concentrations were tested in a range of 0.04–2 mmol/L. Based on the results regarding pH effects, the optimal pH was selected and applied to the different concentrations of the solution, and 0.05 g of the carbonaceous materials (COMAC, AC, or BC) was added to 50 mL Erlenmeyer flasks in 9 cases, and all the flasks were shaken at 210 rpm for 1 h. After filtration, the final metal ion concentrations were measured using ICP-OES.

#### 3.3.3. Adsorption Isotherms

The adsorption isotherm is essential for understanding the interactions between the solution and adsorbents, making it crucial for optimizing the efficiency and effectiveness of adsorbent materials [11]. It is important to note that in adsorption isotherms, the amount of solute adsorbed per unit mass of the adsorbent is expressed as a function of the equilibrium solute concentration, not the initial concentration. Therefore, the model equation can only be used when the equilibrium concentration of the solute is known [34,53].

Two different models were used to fit the adsorption isotherms:

- The Langmuir isotherm model

The Langmuir isotherm model considers the monolayer formation process (e.g., chemical adsorption), where the monolayer adsorption capacity per unit mass of the adsorbent is determined along with the Langmuir constant, which reflects the solute’s affinity for the adsorbent.

The Langmuir isotherm model is expressed by the following non-linear equation:

(3) $$q_{e}=\frac{q_{m\;}K_{L\;}C_{e\;}}{1+K_{L\;}C_{e\;}}$$

where *q_e_* is the equilibrium adsorption amount of heavy metal ions adsorb onto the unit mass (mg/g), *K_L_* is the Langmuir equilibrium constant is related to the energy of adsorption, which quantitatively reflects the affinity between the adsorbent and the adsorbate (L/mg), *C_e_* is the equilibrium ions concentration (mg/L), and *q_m_* is the monolayer adsorption capacity (mg/g).

- The Freundlich isotherm model

The Freundlich isotherm model has been regarded as an empirical equation that describes another multilayer adsorption process, characterized by a gradual increase in the amount of solute adsorbed per unit mass of the adsorbent [10,15,34], and thus, the adsorption occurs on heterogeneous surfaces, where interactions occur between the adsorbed molecules [10].

The non-linear form of the Freundlich model is given by the following equations [33]:

(4) $$q_{e}=K_{f}\;C_{e}^{1/n}$$

where *q_e_* is the amount of heavy metal ions adsorbs onto the unit mass (mg/g), *C_e_* is the equilibrium concentration of adsorbate in the solution (mg/L), *K_f_* is the Freundlich constant, serving as the adsorption or distribution coefficient (it indicates the quantity of metal ions adsorbed per unit of equilibrium concentration on the adsorbent), *n* is the heterogeneity factor (constant factor) that represents the deviation from the linearity of adsorption and provides insight into the favorability of the adsorption process. The value of 1/*n* ranges between 0 and 1. If the value *n* > 1 (1/*n* < 1), adsorption is considered favorable, suggesting a strong interaction between the adsorbent and adsorbate. If *n* = 1, the adsorption is linear, implying uniform adsorption sites, while if *n* < 1 (1/*n* > 1), adsorption is less favorable, indicating that the solvent may have a higher affinity for the adsorbent surface than the adsorbate does [34].

#### 3.3.4. Effect of AC/BC Dose

Adsorbent dosage is also a critical parameter in adsorption, as it determines the amount of metals adsorbed. Since adsorption requires active sites on the adsorbent, a higher dosage of adsorbent increases the number of active sites, making it more effective in removing metals. However, increasing the adsorbent dose even further can reduce the adsorption capacity because more active sites remain unoccupied throughout the process [54].

In this experiment, the effect of adsorbent dose on the uptake of Co^2+^ was investigated using different amounts of 0.05, 0.1, 0.2, 0.3, 0.4, 0.5, 0.6, 0.7, and 0.8 g of the adsorbents. The experiments were carried out by adjusting the pH to the optimal value of 6.8, initial Co^2+^ concentration of 2 mmol/L, and shaking time for 1 h at 210 rpm.

The resulting data were analyzed through curve fitting and statistical methods, all of which were performed using Origin 2021 software.

## 4. Conclusions

AC and BC samples, prepared from *Sargassum* sp. waste, were investigated as materials potentially applicable in heavy metal removal and compared with a commercial activated carbon sample to assess their applicability. The tests were carried out by varying different parameters, including pH, initial concentration, and amount of adsorbents. The adsorption ability of Co^2+^ ions by the AC/BC samples was tested at an initial concentration of 2 mmol/L and 0.05 g at various pH values. As the pH increases, the deprotonation of the metal binding sites increases the negative charge on the surfaces of activated carbon and biochar, which enhances adsorption. However, precipitation could occur above a certain pH, making the optimal pH ~6.8. The pH_pzc_, was determined as well, and it was found that AC has a low pH_pzc_ of 3.40, so its surface remains negatively charged at pH 6.8, which favors the adsorption of Co(II) cations and leads to higher adsorption than other samples.

The results also showed that the adsorption capacity increased with increasing initial concentration for all the adsorbents, reaching the optimum at ~2 mmol/L. The Langmuir and Freundlich isotherm models were applied to Co^2+^ adsorption data. Maximum adsorption capacities determined by the Langmuir model were 468.97, 361.23, and 334.36 mg/g for AC, COMAC, and BC, respectively. The AC sample showed superior adsorption capacities than other samples, indicating that it is more efficient in removing cobalt ions from the solution with adsorption occurring on a homogeneous surface through monolayer adsorption. The K_f_ adsorption capacity in the Freundlich isotherm was higher in AC with 192.53 mg/g compared to other samples. The high surface areas of AC (1695 m^2^/g) significantly enhance the adsorption efficiency by providing abundant active sites. In addition to the large surface area, the negatively charged surface of AC further contributes to its high adsorption capacity. Applying different amounts of the adsorbent with constant pH = 6.8 and an initial metal concentration of 2 mmol/L for all the samples was also carried out. The results showed that AC was the most effective adsorbent for removing Co^2+^ ions compared to COMAC and BC at all dosages, with a removal efficiency of up to 94.4%.

After use, these carbonaceous adsorbents can be regenerated through chemical or thermal treatment to remove the adsorbed contaminants, restoring their functionality, or alternatively managed through disposal methods such as landfilling or incineration. They may also be reused in other applications, including as soil amendments in agriculture or as additives in construction materials. The overall approach improves the environmental sustainability of *Sargassum*-derived materials while also highlighting their potential significance in eco-friendly water treatment systems.

## Figures and Tables

**Table 1:** Comparison of characteristics of the carbonaceous materials COMAC, AC, and BC.

Characteristic of the Samples	Carbonaceous Materials		
	COMAC	AC	BC
Surface area (m^2^/g)	1120 ± 4.5	1695 ± 7	854 ± 4
Zeta potential (mV)	−13.21 ± 0.43	−20.07 ± 1.71	−22.80 ± 0.28
FTIR	Functional group		
	For all samples (-CH, C-O, C=O, O=C=O, C-H, -OH)		

**Table 2:** The adsorbed amount of Co^2+^ ions by the tested samples: COMAC, AC, and BC.

pH	Adsorbed Amount mg/g		
	COMAC	AC	BC
2.8	9.1	13.2	5.7
3.6	9.7	16.6	8.6
4.4	9.8	20.0	9.1
5.2	7.8	21.1	6.1
6.0	9.2	21.1	5.5
6.8	10.8	26.5	7.7
7.6	5.8	27.6	10.5
8.4	15.0	31.7	22.7

**Table 3:** The adsorption isotherm model and the corresponding parameters for Co^2+^ removal by using the studied adsorbents.

Adsorbent	Model						
	Langmuir Model			Freundlich Model			
	q_m_ (mg/g)	K_L_ (L/mg)	R^2^	K_F_ (mg/g)	n	1/n	R^2^
COMAC	361.23	0.01	0.90	7.45	1.35	0.74	0.91
AC	468.97	0.9	0.91	192.53	4.24	0.23	0.90
BC	334.36	0.03	0.90	21.30	1.66	0.60	0.99

**Table 4:** Activated carbon and biochar derived from natural materials and their removal efficiency of potentially toxic metals.

Adsorbent	Toxic Metals	Adsorption Capacity (mg/g)	Reference
AC/Commercial	Co(II)	361.23	*
AC/*Sargassum* sp.	Co(II)	468.97	*
BC/*Sargassum* sp.	Co(II)	334.36	*
BC/coconut fiber	Co(II)	106.8	[28]
BC/Greenhouse Crop Residue	Co(II)	30.98	[29]
AC/Pine cone	Pb(II)	27.5	[40]
AC/Rice Husk	Pb(II)	172.7	[41]
BC/Bamboo	Cd(II)	73.4	[42]
BC/poplar sawdust	Cd(II)	49.3	[43]
BC/Rice straw	Cd(II)	65.4	[44]
AC//Rice Husk	Zn(II)	128.7	[41]
AC/Wheat Straw	Cr(VI)	125.6	[45]
BC/Gingko leaf	Cu (II)	59.9	[46]
BC/cauliflower leaves	Cu (II)	75.9	[47]
AC/Banana Peels	Ni(II)	27.4	[48]

* Current results.

## Data Availability

The original contributions presented in the study are included in the article, further inquiries can be directed to the corresponding author/s.

## Supplementary Materials

The following supporting information can be downloaded at: https://www.mdpi.com/article/10.3390/ijms26167666/s1.

## Abbreviations

The following abbreviations are used in this manuscript:

AC	Activated Carbon
BC	Biochar
COMAC	Commercial Activated Carbon
FTIR	Fourier Transform Infrared Spectroscopy
BET	Brunauer–Emmett–Teller Isotherm
ICP-OES	Inductively Coupled Plasma Optical Emission Spectrometer
pH_pzc_	pH at the point of zero charge
C_e_	equilibrium metal concentration
C_i_	initial metal concentration
K_L_	Langmuir equilibrium constant
q_m_	Maximum monolayer adsorption capacity
K_f_	Freundlich constant
n	heterogeneity factor
