# Supplementary material for: Cobalt Ion Removal by Activated Carbon and Biochar Derived from Sargassum sp
Source: Int J Mol Sci. 2025 Aug 8;26(16):7666. doi: 10.3390/ijms26167666 (PMC12386870; doi:10.3390/ijms26167666)
Supplement: Supplementary file 1 [file ijms-26-07666-s001.zip › ijms-3696635-supplementary.pdf]

Cobalt Ion Removal by Activated Carbon and Biochar Derived from *Sargassum* sp.

Julie Mallouhi [1,2], Emőke Sikora [1,2], Kitti Gráczer [1], Olivér Bánhidi [1], Sarra Gaspard [3], Marckens Francoeur [3], Yeray Alvarez-Galvan [3], Francesca Goudou [3], Béla Viskolcz [1,2], Emma Szőri-Dorogházi [1,2,*] and Béla Fiser [1,2,4,5,*]

1 Institute of Chemistry, University of Miskolc, 3515 Miskolc-Egyetemváros, Hungary
2 Higher Education and Industrial Cooperation Centre, University of Miskolc, 3515 Miskolc-Egyetemváros, Hungary
3 Laboratory COVACHIM-M2E, EA 3592, Université des Antilles, BP 250, 97157 Pointe à Pitre Cedex, France
4 Department of Biology and Chemistry, Ferenc Rakoczi II Transcarpathian Hungarian College of Higher Education, 90200 Beregszász, Ukraine
5 Department of Physical Chemistry, Faculty of Chemistry, University of Lodz, 90-236 Lodz, Poland
* Correspondence: emma.szori-doroghazi@uni-miskolc.hu (E.S.-D.)

Supporting Information

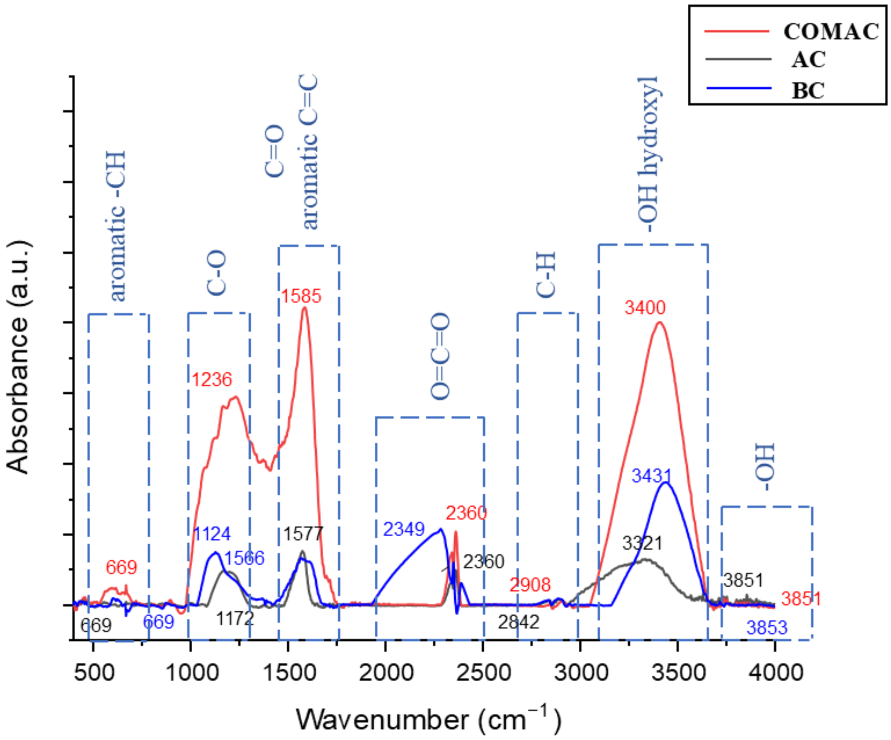

Figure S1. FTIR spectra of the three different activated carbon/biochar (COMAC, AC, and BC) samples
